# Survival and Recovery From Postmyocardial Infarction Apical Wall Rupture With a Complex Course

**DOI:** 10.1155/2024/8888201

**Published:** 2024-10-08

**Authors:** Hussam Al Hennawi, Angad Bedi, Jesse Cheng, Philip Lim, Nawar Al-Rawas, Mauricio Garrido, Aswin Mathew, Jennifer A. Mazzoni

**Affiliations:** ^1^Department of Internal Medicine, Jefferson Abington Hospital, Abington, Pennsylvania, USA; ^2^Department of Pharmacy, Jefferson Abington Hospital, Abington, Pennsylvania, USA; ^3^Department of Radiology, Jefferson Abington Hospital, Abington, Pennsylvania, USA; ^4^Department of Critical Care, Jefferson Abington Hospital, Abington, Pennsylvania, USA; ^5^Department of Cardiothoracic Surgery, Jefferson Abington Hospital, Abington, Pennsylvania, USA; ^6^Department of Cardiology, Thomas Jefferson University Hospital, Philadelphia, Pennsylvania, USA

**Keywords:** cangrelor, left ventricular rupture, pericardial patch, ST-elevation myocardial infarction

## Abstract

Ventricular wall rupture is associated with poor outcomes subsequent to an acute myocardial infarction. We describe a case of postmyocardial infarction apical wall rupture following percutaneous coronary intervention. Our case emphasizes the importance of swift evaluation, diagnosis, and management to enhance survival in individuals confronting this critical condition.

## 1. Introduction

With the introduction of primary percutaneous coronary intervention (PCI), reperfusion therapies have significantly decreased the incidence of mechanical complications, such as left ventricular (LV) free wall ruptures (FWRs) associated with myocardial infarctions (MIs) [1, 2]. Nevertheless, LVFWRs remain among the most lethal mechanical complications following an acute myocardial infarction (AMI) [1]. While recent clinical trials have shown LVFWRs to occur in approximately 0.01%–0.52% of patients after an ST-elevation myocardial infarction (STEMI), this is associated with > 80% risk of in-hospital mortality [1–3]. We report a 57-year-old male who underwent rescue intervention due to an LVFWR subsequent to a STEMI.

## 2. Case Report

**Figure 1:**
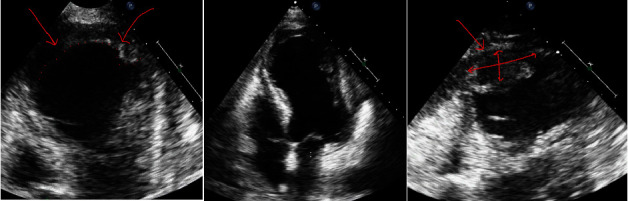
Transthoracic 2D echocardiogram showing severely decreased left ventricular systolic function with global hypokinesis and apical dyskinesis. Large mural layered thrombus in the left ventricular apex. Small to moderate pericardial effusion with no tamponade physiology. Red arrows demonstrate the site of wall rupture.

**Figure 2:**
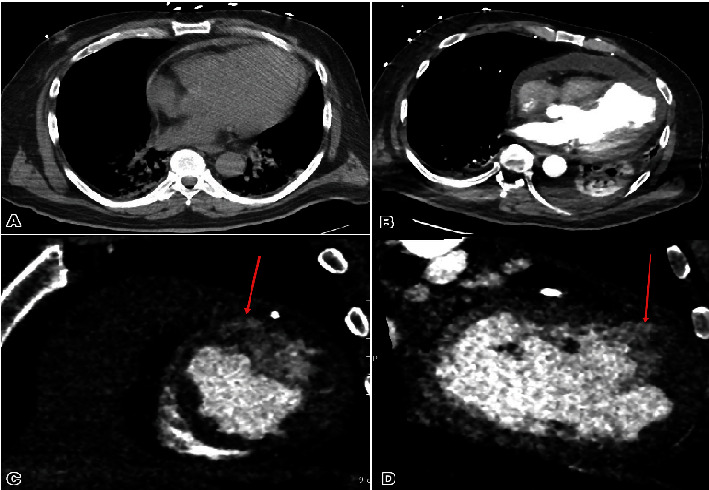
The size of the pericardial effusion has shown progressive enlargement since the previous examination (A) in contrast to (B). Enhancement is observed along the peripheral margins, with a measured width of 21 mm compared to the previous 9 mm. The density remains at 26 Hounsfield units pre- and postcontrast administration. A contrast projection extends to the left ventricle's outer wall at the anterior apical wall, raising concerns for a focal rupture (C, D). Additionally, a left ventricle apical thrombus is present (D). Hypodensity within the left ventricle myocardium suggests a prior myocardial infarction. Red arrows demonstrate the site of wall rupture.

A 57-year-old male patient was transferred from an outside hospital for chest pain and shock. He had a history of poorly controlled diabetes mellitus, hyperlipidemia, and chronic kidney disease. He was recently admitted for a late presentation of STEMI 15 days ago and had undergone placement of three drug-eluting stents in the left anterior descending artery (LAD) and plain old balloon angioplasty of the left circumflex artery (LCX). His ejection fraction was noted to be 20%–25%, and he was ultimately discharged on a life vest. His medications prior to admission included aspirin 81 mg, prasugrel 10 mg, carvedilol 3.125 mg twice daily, and atorvastatin 80 mg. Uptitration of goal-directed medical therapy was limited secondary to systolic hypotension. His blood pressure was 76/52 mmHg, heart rate 104 beats per minute, and regular respiratory rate 20 per minute with appropriate saturations. Jugular venous pressure was normal and had a 2/6 systolic murmur at the left sternal border with a diffuse point of maximal impulse. His lower extremities were warm with trace edema. Due to persistent hypotension, he was started on norepinephrine 0.1 mcg/kg/min. An electrocardiogram (ECG) showed a resolving pattern of ST elevation throughout the lateral leads with interval development of pathological q waves compared to prior ECG. A chest x-ray showed a mild enlargement of the cardiomediastinal silhouette. A transthoracic echocardiogram revealed severely decreased LV systolic function with an estimated ejection fraction of 20%–25% and a large (3.4 × 1.7 cm) mural layered thrombus in the LV apex, with concerns of left ventricle aneurysm versus pseudoaneurysm with small to moderate pericardial effusion with no tamponade physiology (Figure 1). Computerized tomography (CT) of coronary arteries and aorta showed anterior LV wall rupture with contrast noted in the pericardium and a large mural thrombus (Figure 2).

**Figure 3:**
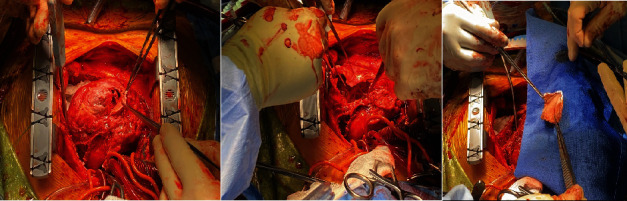
Intraoperative demonstration of left ventricular free wall rupture and patch placement of the left ventricular opening with two layers of the bovine pericardium with felt within the center of it.

Blood work on presentation is as follows: creatinine 1.27 mg/dL (0.7–1.3 mg/dL), high-sensitivity troponin 996 ng/L (< 19 ng/l), NT-proBNP 8834 pg/mL (≤ 125 pg/mL), and lactate 1.7 mmol/L (0.5–2.0 mmol/L). The diagnosis of cardiogenic shock secondary to subacute rupture of the anterior LV was made. Prasugrel was switched to cangrelor and aspirin to preserve LAD patency in anticipation of surgical intervention with no complications. He underwent urgent sternotomy 4 days following his admission, along with resection of infarct territory on the left ventricle and patch placement of the LV opening with two layers of the bovine pericardium with felt within the center of it (Figure 3) along with an elective placement of intra-aortic balloon pump via the right femoral artery which was removed the following day.

His volume status improved with the diuretic regimen, and the patient continued to be hemodynamically stable. The patient was discharged 6 days following the procedure on clopidogrel and warfarin. Secondary to the LV thrombus, the patient is continued on a VitK antagonist. The patient has been followed in the clinic with an unremarkable progression 4 weeks later.

## 3. Comment

FWR, a severe complication of AMI, carries a high in-hospital mortality rate of 80% [4]. Despite its estimated incidence dropping to as low as 0.01% due to the widespread use of reperfusion therapy, the actual occurrence might be higher as a considerable number of patients succumb to sudden death before hospital assessment [3]. Current guidelines of the American College of Cardiology Foundation/American Heart Association and European Society of Cardiology recommend that rapid intervention through emergency surgery is crucial for the survival of patients who present with a window of opportunity for intervention [5, 6].

Diagnosing LVFWR following STEMI involves a multifaceted approach utilizing blood testing, including cardiac biomarkers and inflammatory markers, and imaging studies such as echocardiography, cardiac MRI, and CT scans. These aid in confirming the diagnosis and assessing the extent of the rupture by visualizing signs like pericardial effusion and abnormal wall motion [4]. Risk factors for FWR post-STEMI include advanced age, large infarct size, and delayed reperfusion therapy, while collateral circulation from conditions like hypertension and diabetes may offer some protection [4]. Timely decisions in PCI patients with concurrent FWR are critical, balancing antiplatelet therapy continuation against the need for urgent sternotomy. Cangrelor can safely aid this transition to surgery.

Cangrelor is an intravenous reversible P2Y12 platelet inhibitor that has a half-life of 3–6 min, making it an attractive option for patients with a recent PCI requiring holding oral P2Y12 inhibitors prior to CABG as demonstrated by the BRIDGE trial [7]. In our patient, a DES was placed in the LAD less than a month prior to admission, and the patient was maintained on prasugrel therapy. Upon admission, the patient was bridged with cangrelor at a dose of 0.75 mg/kg/min × actual body weight, with the infusion starting 30 h after the last dose of prasugrel 10 mg. The patient was maintained on this dose for 53 h and shut off 2 h prior to the procedure's start. Cangrelor was then initiated at the same dose 7 h after the end of the procedure and then subsequently transitioned to warfarin and clopidogrel 24 h later with a 600 mg load. Surgical intervention remains the cornerstone of FWR management [5, 6].

## Data Availability

The authors have nothing to report.
